# Supplementary material for: A Likelihood Approach for Real-Time Calibration of Stochastic Compartmental Epidemic Models
Source: PLoS Comput Biol. 2017 Jan 17;13(1):e1005257. doi: 10.1371/journal.pcbi.1005257 (PMC5240920; doi:10.1371/journal.pcbi.1005257)
Supplement: S1 File — (TAR.GZ) [file pcbi.1005257.s014.tar.gz › HSPH_Online-SI-Revision/EnKF07/n10-extreme/n10-extreme_EnKF_table4.pdf]

1	R0	{2.68692, 12.5884}	{2.53346, 10.5038}	{{1.76954, 5.94093}, {3., 13.7354}}	{20.16, 26.7417}	inf	669.	166.674	{71.1454, 229.601}	79.7537	0.76654	newly	306.	106.1	{34., 120.}	75.9412	0.676937	Reff	0.305503	0.491202	{0.135317, 0.768977}	80.9483	ar	9089.	17139.9	{16060., 19695.}	88.5784
2	R0	{1.79802, 12.5412}	{2.45266, 3.88431}	{{1.59543, 2.91764}, {3., 9.70128}}	{45.1485, 69.0276}	inf	841.	881.482	{68.9988, 2271.53}	106.495	0.521494	newly	397.	1341.67	{79., 5753.}	277.992	0.428749	Reff	0.656637	0.581464	{0.126478, 1.46913}	51.8802	ar	7412.	17707.6	{15284., 26128.}	138.904
3	R0	{2.6174, 10.1105}	{1.89602, 2.93476}	{{0.351147, 0.476683}, {3., 3.6704}}	{44.5172, 72.4816}	inf	459.	318.35	{160.198, 417.021}	83.7532	0.450442	newly	256.	378.76	{148., 341.}	98.3125	0.22316	Reff	0.268022	0.278209	{0.0461489, 0.488124}	66.3548	ar	9109.	17318.3	{16226., 18871.}	90.1227
4	R0	{2.27532, 14.3149}	{2.47475, 5.80771}	{{1.54421, 3.58816}, {3., 8.15305}}	{31.8775, 59.4289}	inf	750.	816.303	{73.0536, 281.309}	132.745	0.619361	newly	320.	648.69	{49., 857.}	157.791	0.384146	Reff	0.473267	0.456645	{0.132248, 0.811064}	46.9419	ar	8478.	17516.4	{15351., 22017.}	106.61
5	R0	{2.96649, 18.0674}	{2.4904, 10.3531}	{{1.38515, 0.792978}, {3., 18.2651}}	{17.427, 43.2441}	inf	1193.	247.533	{17.5402, 482.379}	87.0407	1.0699	newly	386.	254.87	{14., 375.}	96.9352	0.757587	Reff	0.251262	0.554293	{0.112087, 1.21367}	139.717	ar	9476.	16155.6	{15130., 17799.}	70.4899
6	R0	{2.94778, 16.0002}	{2.46976, 10.6857}	{{1.81771, 6.01189}, {3., 13.8637}}	{18.1648, 33.215}	inf	1090.	195.92	{49.4971, 218.734}	87.0748	0.955171	newly	362.	114.71	{24., 127.}	79.0801	0.723911	Reff	0.295957	0.498874	{0.163528, 0.813407}	83.2963	ar	9321.	16868.7	{15728., 20046.}	80.9753
7	R0	{2.17562, 11.7184}	{2.08917, 5.69961}	{{0.230362, 0.698231}, {3., 10.4482}}	{54.7486, 51.3619}	inf	793.	721.351	{69.8167, 1328.33}	113.99	0.648566	newly	406.	720.43	{43., 1733.}	152.175	0.50577	Reff	0.496041	0.420451	{0.0450265, 0.818619}	60.103	ar	8345.	17748.3	{16064., 22715.}	112.682
8	R0	{2.7904, 17.8966}	{2.23738, 3.31522}	{{0.643337, 2.69496}, {3., 3.78177}}	{29.5837, 81.4757}	inf	1403.	278.935	{21.8777, 590.431}	83.7899	1.031	newly	475.	384.75	{34., 896.}	89.4084	0.568704	Reff	0.341266	0.486622	{0.0402476, 0.734906}	80.671	ar	9285.	16138.3	{15059., 17123.}	73.8099
9	R0	{2.51574, 16.376}	{2.12592, 7.39796}	{{0.511903, 3.61063}, {3., 11.1853}}	{40.1341, 54.8244}	inf	1116.	297.286	{107.224, 399.19}	82.768	0.739501	newly	427.	277.07	{80., 452.}	68.808	0.450162	Reff	0.397235	0.456423	{0.0694902, 0.716795}	60.3872	ar	8937.	15868.2	{15174., 16514.}	77.5565
10	R0	{2.56405, 18.1787}	{2.20118, 7.43752}	{{0.763871, 3.60431}, {3., 11.4272}}	{35.2354, 59.0866}	inf	1437.	524.048	{223.811, 691.468}	81.3271	0.689709	newly	467.	898.67	{114., 891.}	184.413	0.480144	Reff	0.441786	0.561289	{0.0488284, 0.790641}	89.4262	ar	9005.	16931.1	{15438., 17762.}	88.0191
11	R0	{2.475, 17.1709}	{2.6113, 6.13762}	{{1.65432, 0.603834}, {3., 17.4233}}	{22.0688, 65.5411}	inf	1270.	1061.76	{466.618, 2277.18}	70.1813	0.372634	newly	447.	1590.68	{213., 1997.}	284.908	0.427024	Reff	0.449708	0.499876	{0.0112068, 1.16156}	65.9725	ar	8870.	17195.5	{14968., 20529.}	93.8608
12	R0	{2.14974, 11.8619}	{2.17398, 7.38668}	{{0.768893, 3.59702}, {3., 9.61235}}	{49.2513, 37.7277}	inf	994.	594.984	{201.282, 912.281}	83.9376	0.556251	newly	494.	949.17	{145., 769.}	189.751	0.487209	Reff	0.487776	0.567816	{0.0364574, 0.796853}	73.9461	ar	8414.	17151.	{15545., 18281.}	103.839
13	R0	{2.20385, 14.4767}	{2.71712, 10.0842}	{{2.05707, 6.32387}, {3., 15.4057}}	{27.2858, 34.9861}	inf	760.	584.976	{245.701, 489.34}	86.294	0.418637	newly	319.	337.16	{102., 365.}	63.1536	0.244117	Reff	0.478235	0.669284	{0.289211, 0.716061}	59.0344	ar	8347.	16862.6	{15443., 19822.}	102.02
14	R0	{2.35671, 13.8357}	{2.37272, 9.89187}	{{1.09596, 0.684612}, {3., 18.7923}}	{35.8975, 64.1034}	inf	848.	197.674	{23.219, 310.459}	84.4107	0.954316	newly	361.	271.24	{14., 588.}	97.1357	0.719453	Reff	0.411717	0.654731	{0.159394, 1.09835}	81.4229	ar	8719.	16423.3	{15424., 18307.}	88.3621
15	R0	{1.91368, 10.1082}	{2.47929, 5.94485}	{{1.55267, 2.60701}, {3., 8.44794}}	{43.1044, 41.1878}	inf	436.	305.688	{30.4503, 338.516}	107.766	0.721691	newly	261.	430.45	{20., 372.}	178.51	0.613179	Reff	0.581759	0.507261	{0.171611, 0.976137}	48.087	ar	7486.	16629.9	{15219., 18849.}	122.146
16	R0	{2.48727, 14.882}	{2.42744, 9.82062}	{{1.1512, 0.834432}, {3., 19.2726}}	{30.853, 62.0327}	inf	1044.	386.914	{135.969, 657.233}	72.8377	0.596639	newly	425.	527.64	{71., 1013.}	103.581	0.444892	Reff	0.402192	0.628665	{0.118661, 1.15292}	84.6409	ar	8894.	16573.6	{15356., 19387.}	86.3459
17	R0	{2.46795, 13.1915}	{2.08126, 6.04335}	{{0.204372, 0.616446}, {3., 9.97699}}	{44.5566, 54.1875}	inf	950.	625.806	{77.5597, 985.672}	100.144	0.682223	newly	457.	675.11	{38., 1371.}	143.298	0.611951	Reff	0.428189	0.363013	{0.0103913, 0.745867}	61.0026	ar	8796.	17559.2	{16007., 19773.}	99.6265
18	R0	{2.96603, 16.7543}	{2.5695, 9.71866}	{{1.44359, 0.720106}, {3., 18.0902}}	{14.9036, 49.3372}	inf	1079.	179.425	{21.3031, 460.06}	83.8185	1.05275	newly	402.	394.33	{17., 350.}	133.4	0.779218	Reff	0.284146	0.59096	{0.15125, 1.1492}	126.078	ar	9394.	16254.1	{15239., 18179.}	73.0267
19	R0	{2.25216, 9.90465}	{2.72387, 6.19388}	{{2.27334, 0.980035}, {3., 9.53379}}	{20.9447, 37.465}	inf	643.	512.291	{57.2211, 648.548}	95.1973	0.566147	newly	379.	888.53	{41., 766.}	220.953	0.549515	Reff	0.403812	0.585824	{0.204322, 0.927455}	73.6013	ar	8631.	18467.9	{16599., 21141.}	113.971
20	R0	{2.70837, 18.1923}	{2.17815, 7.27694}	{{0.563886, 3.61484}, {3., 10.939}}	{33.5751, 59.9999}	inf	1399.	255.841	{47.6182, 457.069}	81.7126	0.845723	newly	468.	242.74	{49., 564.}	60.265	0.511924	Reff	0.365088	0.46115	{0.0849466, 0.704738}	64.3588	ar	9183.	16022.1	{15373., 16617.}	74.4753
21	R0	{2.30288, 15.5877}	{1.89198, 6.35433}	{{0.467918, 0.979164}, {3., 10.327}}	{51.7469, 59.235}	inf	1029.	805.046	{100.42, 2386.69}	86.3139	0.517416	newly	405.	610.05	{66., 1368.}	104.21	0.374882	Reff	0.441923	0.318112	{0.105691, 0.685982}	58.2569	ar	8674.	17653.4	{15610., 21827.}	103.521
22	R0	{2.82208, 10.7544}	{1.73166, 5.88536}	{{0.389251, 3.691}, {3., 7.68075}}	{44.5652, 45.2748}	inf	586.	391.784	{207.222, 581.128}	51.6633	0.297714	newly	278.	333.84	{176., 630.}	60.6475	0.203189	Reff	0.244392	0.247727	{0.064325, 0.514466}	73.9381	ar	9333.	18135.5	{17158., 20881.}	94.3157
23	R0	{1.69923, 10.1366}	{2.69114, 10.7969}	{{2.1214, 6.91744}, {3., 16.6515}}	{58.3739, 44.623}	inf	558.	434.364	{35.613, 483.837}	89.8036	0.558333	newly	330.	324.36	{21., 385.}	100.588	0.599343	Reff	0.599318	0.524473	{0.118409, 0.771548}	47.4073	ar	7055.	16617.6	{15052., 17291.}	135.544
24	R0	{2.64944, 19.054}	{1.92884, 6.39204}	{{0.503244, 1.00931}, {3., 10.3709}}	{42.2822, 66.453}	inf	1099.	717.639	{45.248, 1843.31}	88.8706	0.687747	newly	382.	536.73	{33., 1115.}	107.704	0.503359	Reff	0.35317	0.357044	{0.0814356, 0.540381}	50.0988	ar	9147.	17250.5	{15423., 21541.}	88.5916
25	R0	{2.53191, 10.9214}	{1.96868, 6.96568}	{{0.618417, 3.62641}, {3., 11.047}}	{41.1026, 37.2549}	inf	571.	328.343	{95.2794, 554.598}	54.2788	0.399276	newly	309.	350.27	{84., 565.}	79.9709	0.335558	Reff	0.310159	0.469824	{0.064494, 0.72271}	104.309	ar	9009.	16574.6	{15114., 20587.}	83.9785
26	R0	{1.89155, 6.76944}	{2.44088, 5.86491}	{{1.30545, 0.810963}, {3., 10.0018}}	{41.0474, 65.7671}	inf	234.	364.577	{35.2653, 466.275}	149.678	0.502802	newly	188.	700.77	{21., 505.}	358.484	0.603978	Reff	0.540416	0.417123	{0.0656298, 0.850442}	58.3138	ar	7348.	17397.4	{16042., 19146.}	136.764
27	R0	{2.63149, 15.6979}	{2.4404, 10.3875}	{{1.18982, 0.848633}, {3., 19.2858}}	{26.5869, 56.8837}	inf	1228.	390.501	{128.367, 576.928}	74.8961	0.642255	newly	463.	489.08	{80., 948.}	93.3218	0.447726	Reff	0.367882	0.548381	{0.14758, 1.09992}	85.9624	ar	9104.	16442.4	{15267., 18531.}	80.6063
28	R0	{2.85141, 12.6992}	{1.87681, 2.85537}	{{0.499919, 0.801155}, {3., 3.67894}}	{39.5991, 77.5154}	inf	776.	341.776	{150.652, 570.2}	85.1812	0.662755	newly	347.	348.4	{143., 550.}	87.389	0.341819	Reff	0.251494	0.252341	{0.0495968, 0.479443}	63.7119	ar	9317.	17387.3	{16161., 19873.}	86.6191
29	R0	{2.54244, 12.9085}	{2.75287, 3.24374}	{{2.32101, 2.95323}, {3., 3.33329}}	{13.6795, 74.8713}	inf	779.	356.879	{18.1153, 384.324}	92.0897	0.827755	newly	343.	635.04	{26., 1501.}	160.968	0.515777	Reff	0.329754	0.685916	{0.240274, 1.95104}	122.37	ar	9034.	17158.5	{15120., 24063.}	89.9321
30	R0	{2.10982, 11.7636}	{2.74201, 3.59407}	{{2.28648, 0.881556}, {3., 5.645}}	{29.9641, 69.4476}	inf	703.	499.279	{64.9998, 413.946}	107.674	0.664758	newly	366.	868.97	{61., 1513.}	209.232	0.448748	Reff	0.486735	0.55531	{0.130554, 0.943751}	60.5433	ar	8224.	17076.7	{15390., 20056.}	107.644
31	R0	{2.76312, 15.5396}	{2.52677, 10.5728}	{{1.89601, 6.0843}, {3., 14.0074}}	{17.9838, 31.9621}	inf	1113.	212.449	{32.8946, 233.115}	87.8599	0.971922	newly	439.	127.62	{20., 134.}	83.7631	0.807051	Reff	0.324667	0.527697	{0.161421, 0.863236}	82.9002	ar	9220.	17169.6	{15888., 21672.}	86.2213
32	R0	{2.21727, 15.5439}	{2.42342, 6.11818}	{{1.45294, 3.44133}, {3., 6.75994}}	{37.3003, 63.1076}	inf	936.	627.797	{383.878, 631.368}	68.2329	0.361302	newly	399.	630.1	{245., 1050.}	97.0827	0.243413	Reff	0.506203	0.441489	{0.160414, 0.827808}	44.7921	ar	8359.	16292.3	{15129., 18918.}	94.9078
33	R0	{2.72912, 10.8159}	{2.52308, 9.11037}	{{1.74323, 5.19949}, {3., 11.9146}}	{19.8575, 27.4929}	inf	395.	488.939	{107.297, 777.491}	107.867	0.383922	newly	210.	337.3	{66., 407.}	116.886	0.317525	Reff	0.252989	0.60746	{0.311889, 1.2759}	144.962	ar	9196.	18649.5	{16712., 23833.}	102.8
34	R0	{2.20928, 10.1308}	{1.83397, 6.94596}	{{0.450081, 2.23807}, {3., 13.2054}}	{55.6421, 52.9676}	inf	446.	738.435	{26.0436, 2076.73}	147.43	0.544649	newly	238.	497.26	{25., 1148.}	179.185	0.49766	Reff	0.406066	0.238689	{0.0392879, 0.518338}	61.2815	ar	8388.	17911.3	{16476., 22494.}	113.535
35	R0	{2.10103, 15.2673}	{2.74904, 10.9334}	{{2.1833, 7.06345}, {3., 16.3039}}	{30.8426, 33.9453}	inf	986.	524.672	{99.4613, 786.965}	90.4219	0.648951	newly	382.	376.6	{46., 595.}	107.691	0.52013	Reff	0.508869	0.571169	{0.298363, 0.825441}	35.8168	ar	8264.	16759.7	{15458., 17467.}	102.803
36	R0	{2.74914, 19.2775}	{1.93961, 6.39931}	{{0.463142, 0.973201}, {3., 10.5995}}	{40.0315, 66.8042}	inf	1441.	826.199	{123.512, 2622.12}	77.6822	0.592867	newly	436.	589.45	{64., 1515.}	91.094	0.374304	Reff	0.366735	0.337829	{0.0771873, 0.63679}	58.1731	ar	9301.	17680.7	{15459., 22816.}	90.0949
37	R0	{2.78161, 16.0136}	{2.02808, 7.33422}	{{0.154878, 3.60805}, {3., 11.2125}}	{36.9821, 54.2}	inf	1033.	409.369	{156.054, 508.868}	75.7576	0.573262	newly	388.	450.73	{100., 569.}	101.353	0.370951	Reff	0.3146	0.403302	{0.00912914, 0.697566}	92.2782	ar	9240.	16272.5	{15209., 17626.}	76.1097
38	R0	{2.4454, 8.33501}	{2.18655, 5.8909}	{{0.289755, 0.901021}, {3., 9.36452}}	{35.079, 43.7461}	inf	243.	327.56	{44.3803, 331.646}	121.132	0.416529	newly	147.	451.38	{29., 565.}	282.517	0.468178	Reff	0.306653	0.284507	{0.0252415, 0.514457}	53.2439	ar	8853.	17486.3	{16482., 18974.}	97.5181
39	R0	{2.9154, 12.3861}	{2.37777, 6.02701}	{{1.9889, 1.14029}, {3., 9.87132}}	{21.3429, 51.3406}	inf	596.	310.706	{41.902, 470.203}	78.8541	0.576123	newly	284.	629.7	{57., 553.}	197.873	0.427708	Reff	0.225069	0.577045	{0.17254, 1.00722}	169.07	ar	9371.	18420.	{16753., 21613.}	96.5633
40	R0	{2.40923, 15.1963}	{2.0832, 7.21969}	{{0.5755, 3.59282}, {3., 11.1488}}	{43.9387, 52.4905}	inf	1025.	347.135	{155.665, 608.042}	71.2211	0.620147	newly	421.	382.23	{106., 699.}	80.0879	0.436376	Reff	0.424988	0.431063	{0.0904143, 0.77418}	65.7587	ar	8735.	15991.6	{15195., 16791.}	83.0746
41	R0	{2.63137, 15.238}	{2.7763, 10.4334}	{{2.29266, 7.00218}, {3., 14.1704}}	{12.984, 31.5307}	inf	1020.	333.837	{47.7631, 467.374}	86.6516	0.782831	newly	409.	279.03	{36., 360.}	97.	0.625917	Reff	0.336026	0.716306	{0.109605, 1.91572}	142.056	ar	9150.	16944.6	{15270., 22970.}	85.1873
42	R0	{2.48769, 19.4593}	{2.35491, 5.9503}	{{1.3537, 0.263107}, {3., 10.625}}	{30.8738, 69.4218}	inf	1184.	512.082	{57.2463, 1230.07}	74.4099	0.715801	newly	394.	451.14	{28., 916.}	76.8122	0.459435	Reff	0.413205	0.459012	{0.0864025, 1.12941}	73.6142	ar	8932.	17282.5	{15589., 20409.}	93.4896
43	R0	{2.61199, 10.214}	{1.7871, 6.02929}	{{0.483454, 3.69956}, {3., 7.78681}}	{46.1388, 40.9703}	inf	551.	348.359	{159.542, 613.849}	47.1824	0.309199	newly	296.	284.88	{122., 552.}	47.7162	0.230873	Reff	0.299073	0.266002	{0.0785187, 0.517152}	57.3264	ar	9061.	18374.1	{17343., 20498.}	102.782
44	R0	{2.50994, 18.3382}	{2.65034, 9.39409}	{{1.9517, 4.1952}, {3., 12.3424}}	{19.7884, 48.7731}	inf	1257.	602.72	{41.4147, 1342.12}	74.5825	0.670365	newly	414.	409.15	{30., 1025.}	87.6401	0.493723	Reff	0.47714	0.547338	{0.0348601, 0.920288}	66.7796	ar	8827.	16221.9	{13929., 22595.}	83.7755
45	R0	{2.44283, 16.8338}	{2.77071, 10.7761}	{{2.29765, 7.00277}, {3., 16.2307}}	{16.7503, 35.9852}	inf	1098.	442.161	{116.354, 496.566}	85.9243	0.686045	newly	418.	354.12	{44., 448.}	96.1292	0.532427	Reff	0.427251	0.606575	{0.139037, 0.982827}	69.5397	ar	8878.	16963.7	{15534., 17667.}	91.0758
46	R0	{2.51815, 18.1462}	{1.92111, 6.47838}	{{0.539075, 0.985609}, {3., 10.5549}}	{45.1409, 64.299}	inf	1305.	960.344	{120.162, 3136.04}	86.0546	0.58871	newly	464.	711.7	{62., 1753.}	108.444	0.421526	Reff	0.458051	0.327468	{0.0930362, 0.653403}	55.7554	ar	8978.	17769.7	{15396., 23107.}	97.9247
47	R0	{2.88885, 16.8987}	{2.7525, 3.6618}	{{2.25249, 0.798795}, {3., 5.75262}}	{9.64473, 78.3309}	inf	1290.	544.511	{25.7909, 571.666}	96.0261	0.936101	newly	468.	945.74	{31., 2587.}	189.222	0.589164	Reff	0.329618	0.573799	{0.0814293, 1.31668}	103.609	ar	9337.	17329.5	{15531., 22390.}	85.6007
48	R0	{2.58709, 13.8786}	{2.47176, 10.8391}	{{1.82247, 6.07286}, {3., 14.209}}	{22.0142, 24.2187}	inf	1037.	167.89	{70.5688, 234.407}	85.647	0.971587	newly	435.	99.03	{25., 129.}	79.7908	0.84517	Reff	0.330113	0.509682	{0.204231, 0.771811}	69.8319	ar	9146.	16675.6	{15546., 19320.}	82.3264
49	R0	{2.42959, 10.7132}	{2.68081, 5.8006}	{{2.27456, 1.03297}, {3., 9.25804}}	{15.955, 45.8556}	inf	676.	632.98	{73.2323, 1072.41}	112.434	0.603302	newly	359.	930.79	{49., 1100.}	237.68	0.552868	Reff	0.358122	0.545749	{0.19898, 0.954809}	79.3497	ar	8863.	18708.8	{16671., 22788.}	111.089
50	R0	{2.60513, 13.2796}	{2.49379, 10.333}	{{1.32555, 0.949157}, {3., 19.4829}}	{25.1511, 68.6178}	inf	1051.	464.601	{154.157, 875.327}	64.2248	0.494499	newly	465.	588.39	{89., 799.}	104.247	0.422214	Reff	0.354037	0.596471	{0.140934, 1.33207}	102.49	ar	9095.	16688.7	{15258., 20976.}	83.4933
